# Acute effect of school-based active breaks on physical activity level and on-task classroom behavior in primary schoolchildren

**DOI:** 10.3389/fpubh.2025.1644819

**Published:** 2025-10-30

**Authors:** Tomás Reyes-Amigo, Alberto Grao-Cruces, David Sanchez-Oliva, Antonio Garcia-Hermoso, Daniel Reyes-Molina, Rodrigo Yañez-Sepúlveda, Jorge Olivares-Arancibia, Juan Hurtado-Almonácid, Jacqueline Páez-Herrera, Gabriel Salinas-Gallardo, Edgardo Mendoza, Camilo Ovalle-Fernández, Felipe Sepúlveda-Figueroa, Jessica Ibarra-Mora

**Affiliations:** ^1^Observatorio de Ciencias de la Actividad Física (OCAF), Departamento de Ciencias de la Actividad Física, Universidad de Playa Ancha, Valparaíso, Chile; ^2^GALENO Grupo de Investigación, Departamento de Educación Física, Facultad de Ciencias de la Educación, Universidad de Cádiz, Puerto Real, Spain; ^3^ACAFYDE Grupo de Investigación, Departamento de Didáctica Musical, Plastica y Expresión Corporal, Facultad de Ciencias del Deporte, Universidad de Extremadura, Caceres, Spain; ^4^Navarrabiomed, Hospital Universitario de Navarra, Universidad Pública de Navarra (UPNA), IdiSNA, Pamplona, Spain; ^5^Escuela de Kinesiología, Facultad de Salud, Universidad Santo Tomás, Los Ángeles, Chile; ^6^Facultad de Educación y Ciencias Sociales, Universidad Andrés Bello, Viña del Mar, Chile; ^7^Escuela de Medicina, Universidad Espíritu Santo, Samborondón, Ecuador; ^8^Grupo AFySE, Investigación en Actividad Física y Salud Escolar, Escuela de Pedagogía en Educación Física, Facultad de Educación, Universidad de las Américas, Santiago, Chile; ^9^Escuela de Educación Física, Pontificia Universidad Católica de Valparaíso, Viña del Mar, Chile; ^10^Departamento de Educación Física, Universidad Metropolitana de las Ciencias de la Educación, Ñuñoa, Chile

**Keywords:** classroom, cognition, physical exercise, active learning, childhood

## Abstract

**Introduction:**

Active breaks (ABs) in the classroom are a promising way to promote children’s active behaviors while contributing to the development of their physical, academic, and cognitive skills. However, the effects of ABs, which are exclusive to classroom settings, remain unclear. The aim of this study was to determine the acute effect of an ABs intervention on physical activity levels and on-task classroom behavior in schoolchildren.

**Method:**

The participants included 55 primary schoolchildren aged between 10 and 11 years (10.48 ± 0.5 years). Children were randomized into an experimental group (EG) and a control group (CG). In the EG, six ABs of 4 min and 30 s were applied during the school day. The CG followed their regular school day. Physical activity levels were assessed throughout the school day using accelerometers (ActiGraph wGT3X-BT, Ametris, United States), and on-task classroom behavior was evaluated using the Direct Behavior Rating Scale.

**Results:**

The EG showed significant differences in the min of physical activity level across all five levels compared to the CG: Sedentary time was significantly lower in the EG [EG 229.83 ± 17.17 vs. CG 253.76 ± 12.81 min, *p* = 0.001; effect size (ES) = −158], while light physical activity level (EG 36.65 ± 11.66 vs. CG 32.20 ± 7.77 min, *p* = 0.002; ES = 1.04), moderate physical activity level (EG 8.78 ± 2.98 vs. CG 7.11 ± 1.81 min, *p* = 0.002; ES = 1.05), vigorous physical activity level (EG 14.76 ± 4.83 vs. CG 6.52 ± 3.23 min, *p* = 0.001; ES = 2.64), and moderate-vigorous physical activity level (EG 23.53 ± 7.12 vs. CG 13.71 ± 4.7 min, *p* = 0.001; ES = 2.18) were all significantly higher. Regarding on-task classroom behavior outcomes, both academic engagement (67.51% ± 25.61 vs. 82.91% ± 18.81; *p* = 0.002; ES = 0.1) and disruption (15.81 ± 17.21% vs. 7.51% ± 14.81 *p* = 0.002; ES = 0.5) showed statistically significant differences before and after the ABs. Regarding respectfulness (84.21% ± 17.41 vs. 90.41% ± 14; *p* = 0.21), the ABs showed no significant changes.

**Conclusion:**

ABs are an effective strategy to acutely increase primary school children’s moderate and vigorous physical activity engagement and improve on-task classroom behavior. Implementation should be considered by policymakers, educators, and health professionals.

**Clinical trial registration:**

ClinicalTrials.gov, identifier NCT05403996.

## 1 Introduction

The benefits of physical activity (PA) on the quality of life of children and adolescents are well established, as it reduces the risk of chronic diseases and the symptoms of depression and anxiety, as well as improves physical fitness, cognitive function, and self-esteem (1–3). Additionally, PA is associated with better academic performance (4, 5) and behavior during school tasks (6–8). Despite this, worldwide reports reveal that more than 80% of children do not reach the recommended levels of PA (9). Moreover, the rates of PA implementation are expected to decrease as children age from the first years of elementary school (10), worsening the physical inactivity status during the growth and development phases. Therefore, the integration of PA into the school routine is a key aspect of reducing physical inactivity behaviors (11). In this sense, increasing the level of PA, especially at moderate-to-vigorous intensity, has significant implications for health and academic performance (12). Elementary schools can be ideal settings for PA in children because of the amount of time spent at school and because they provide a safe environment with educational professionals who can guide not only PA practice but also include educational content (13). However, assigning more time for PA during the school day often conflicts with curricular demands, undermining physical activity and promotion policies (14). Therefore, in order to make PA a priority in the school context, efficient strategies are needed (15, 16). In this regard, active breaks (ABs) are considered an emerging and suitable trend for PA integration into educational settings, fitting the curricular timetable by interspersing extended periods of sitting with brief bouts of PA (17). It has been reported that ABs are effective for increasing PA levels and improving classroom behavior, particularly on-task behavior (18, 19). In this regard, several factors influence the practical implementation of ABs. Evidence indicates that teachers generally have positive perceptions of the use of ABs (15, 20). Notwithstanding, it must be short, fast, suitable to be performed in the limited space available in the classroom, easy to implement (without sophisticated technological equipment), and must not imply a great time responsibility related to the teachers’ academic load (20). Without these characteristics, ABs can have an adverse effect, particularly on classroom behavior (21).

Different reviews (13, 22, 23) proposed two approaches to implementing ABs: (1) ABs as an interval/rest between two successive lessons and (2) ABs taken during the lesson. However, these alternatives have been shown to be limited due to a very broad school curriculum and the established priority for standardized tests (24). Despite these limitations, interventions with ABs implemented by the classroom teacher, or even using basic technology (e.g., audio) (25, 26) for 10 to 20 min, two or three times a week, twice a day (27, 28), or from 3 to 5 min every day (24, 29), have shown effectiveness in improving PA level, academic performance, enjoyment, desire to learn, concentration, and on-task behavior. Therefore, including PA in daily instruction does not detract from academic performance but may enhance it (23). In this sense, this study contributes to the existing gap regarding the incorporation and implementation of PA interventions in the classroom, thereby helping to increase PA levels and on-task behavior. It should be noted that one of the most important aspects of learning is behavior on school tasks, as it has been demonstrated that extended periods of instruction without breaks are detrimental to students’ academic behavior (30). In relation to this, it was discovered that implementing a 10-min classroom ABs increased elementary school pupils’ time on task by 8% immediately (31). In this sense, other studies (19, 32) show that participation in ABs is linked to a rise in elementary school-aged children’s on-task behavior in the classroom. Another interesting result indicates that a brief period of PA in the classroom can help teachers increase time on-task during classes, particularly for the most off-task students (33). However, there is no consensus regarding the type of ABs (e.g., time, intensity, activities) intervention in the school context; therefore, more experimental evidence on the acute effect is required in order to address different strategies (13, 34). The aim of this study was to determine the acute effect of an ABs intervention on PA levels and on-task classroom behavior in schoolchildren.

## 2 Materials and methods

### 2.1 Study design

**Figure 1:**
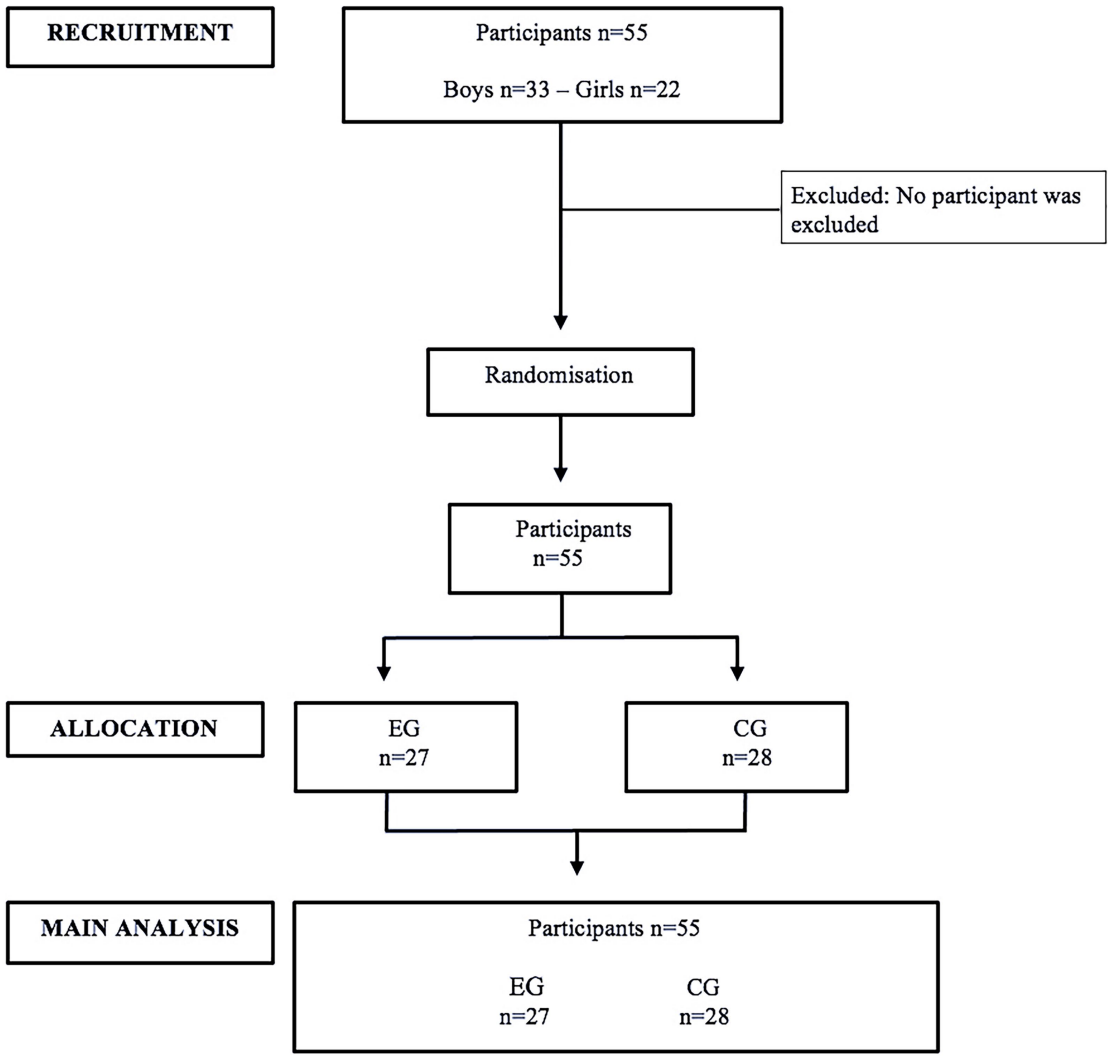
CONSORT participants flow during the study. EG, experimental group; CG, control group.

The study was carried out under a quantitative paradigm. This study used a quasi-experimental design (35). The sample was randomized into two groups: experimental group (EG) and control group (CG). The EG carried out an ABs from the ACTIVA-MENTE program (36) and applied it in their respective classes, while the CG followed their regular school day (without any intervention or extra PA). The protocol was developed in agreement with the Standard Protocol Items: Recommendations for Interventional Trials (SPIRIT) (37) and according to the verification guidelines of the Consolidated Standards of Reporting Trials (CONSORT) (38). Figure 1 provides a general description of the chronogram of enrollment, interventions, and assessments.

### 2.2 Participants

A total of 55 children (33 boys and 22 girls) volunteered to participate in this study. Eligible school leaders were initially invited to participate via e-mail and then contacted a week later by telephone. A researcher met with all interested school leaders to explain the requirements for participation in the study. Recruitment was conducted for 2 months during the first semester of 2024. Finally, the study setting comprised two primary schools in Valparaíso, Chile. The participants were students from low to middle socioeconomic levels (C_3_-D). The age of the students ranged from 10 to 11 years old (10.48 ± 0.5 years), with a sexual maturity stage of Tanner 1–2 (39). Written informed consent from parents or guardians and assent from children were obtained prior to the start of the intervention. Ethical approval for this study was granted by the Ethical Scientific Committee of Playa Ancha University (N°005/2022). The inclusion criteria were students aged 10–11 years belonging to the sixth grade of elementary school. The excluded students were those who presented health impediments when ABs were performed and those with cognitive disabilities. These criteria were used because the effect of the intervention on the study variables depended on the physical and psychological disposition for carrying out the ABs of the students. Participants who met the exclusion criteria were excluded only from the data analysis; those who failed to attend the first or second measurement and/or presented a negative response to make ABs were also excluded. All remaining participants completed the study, and there were no negative effects after the interventions. Therefore, the initial sample was analyzed using the following steps. Schools were randomly assigned through a simple randomization procedure (40), with the sample divided into two groups: EG and CG. As a result of the randomization process, the EG consisted of 27 children (18 boys and 9 girls), and the CG consisted of 28 children (15 boys and 13 girls) (40) (Figure 1). Students who attend these schools spend approximately 8 hours a day at school (from 8:00 a.m. to 4:00 p.m.) with two recesses of approximately 15 min each (30 min altogether) and a longer rest for lunch between 45 min and 1 h. For reasons of viability, the plan was to recruit two schools (one for intervention and one for control purposes). Prior to starting the present study, a power analysis was performed (G*Power 3; Heinrich-Heine-Universität Düsseldorf, Germany) to calculate the adequate sample size (*F*-test, effect size = 0.25, *α* error = 0.05, power = 0.95) (41). Based on this calculation, the participation of 54 students was estimated in this study. This sample size provided sufficient statistical power and can be considered representative of the population (42).

### 2.3 Measurements

The measurements were taken on 2 days, one for PA levels and the other for on-task classroom behavior, both days without physical education.

PA levels: ActiGraph wGT3X-BT accelerometers and ActiLife 6 (United States) software were used to measure the children’s PA levels. This device has proven validity and reliability in objectively measuring PA levels in children (43–45). PA levels were monitored only on school days. Accelerometers were used on the dominant wrist during school days in both EG and CG. Three study collaborators distributed the accelerometers and picked them up from the children at school at the beginning and end of each school day. A short-duration epoch of 15 s was used because the pattern was more intermittent in children’s PA levels (46) with frequencies of 100 Hz. The classification of the daily PA level was based on sedentary time, light, moderate, and vigorous PA, established from counts per min. The cut-off point used was determined by Evenson’s equation for the ActiGraph wGT3X-BT, especially used for students’ tasks, and classroom behavior (47) (sedentary = 0–100 counts × min^−1^, light = 100–2,295 counts × min^−1^, moderate = 2,296–4,012 counts × min^−1^, vigorous = 4,013 counts × min^−1^).

On-task classroom behavior: Information was compiled on the behavior of the students in the classroom individually using the Direct Behavior Rating Scale (48, 49) for universal use (50). The Direct Behavior Rating Scale is a data collection technique for student behavior that combines a rating scale approach with direct observation. This scale assesses three core behavioral competencies that are critical to student success: *academically engaged* (actively or passively participating in the classroom activity, for example: writing, raising hand, answering a question, talking about a lesson, listening to the teacher, reading silently, or looking at instructional materials); *respectful* (defined as compliant and polite behavior in response to adult direction and/or interactions with peers and adults, for example: follows teacher direction, pro-social interaction with peers, positive response to adult request, verbal or physical disruption without a negative tone/connotation); and *disruptive behavior* (student action that interrupts regular school or classroom activity, for example: out of seat, fidgeting, playing with objects, acting aggressively, talking/yelling about things that are unrelated to classroom instruction). This observation tool required the teacher to indicate for each child, on a scale from 0 (Never = 0%) to 5 (Sometimes = 50%) to 10 (Always = 100%), in relation to the percentage of time they spent on the task, i.e., how committed they were to the task (e.g., listening to the teacher, writing, looking at instruction materials) during the observation period. The observation was conducted on one school day in the EG and CG. Twelve members of the research team, trained in the use of this instrument, were assigned to each participant to conduct the observations. They then conducted individualized behavioral observations of the students for 15 min before and after the administration of ABs to establish the acute effects of these. A test–retest procedure was conducted to assess the inter-observer agreement.

The assessment was carried out by a group of collaborators properly trained by the research team in four sessions prior to the application of the instruments. The evaluators were not familiar with the students in the study. They only performed data collection (Table 1).

**Table 1:** Procedure data collection.

Outcomes	Group and day measurement						
	EG/day 1		EG/day 2			CG/day 1	
Physical activity level	Install Acc: start of the school day	Remove Acc: end of school day				Install Acc: start of the school day	Remove Acc: end of school day
On-task classroom behavior			Obs. DBR: Pre-ABs	ABs	Obs. DBR: Pos-ABs		

EG, experimental group; CG, control group; ABs, active-break; Acc, accelerometer; DBR, direct behavior rating.

### 2.4 Intervention

The single ABs intervention corresponded to the ACTIVA-MENTE Program (36). Activities in this program are presented via videos, so only the sound and projection equipment available in schools was required. The intervention consisted of teachers applying ABs through previously recorded videos that were specially designed for the program. The program’s official website is freely accessible online: https://convivenciaparaciudadania.mineduc.cl/activamente/. These videos last 4 min and 30 s. This time is divided into 1 min of preparation (general explanation and indications) and 3 min for six activities (e.g., jumps with feet together, skipping, jumping jacks, and scissor kicks) of moderate to high intensity. The intensity was monitored using the rated perceived exertion (RPE) scale (51). Each activity was performed for 20 s, followed by 10 s of recovery (52). During the recovery period, the following activity was explained: the final 30 s were for the cool-down (Table 2). The general guidelines of the intervention ACTIVA-MENTE program can also be found at the following link: https://bibliotecadigital.mineduc.cl/handle/20.500.12365/17520.

**Table 2:** Description of ABs session (ACTIVA-MENTE program).

Groups	Indications	Activities description	Times
EG	In-person classes: students beside their desks	Beginning: general instructions	1 min
	Teacher’s instructions to the class	Physical activity: six activities	3 min total
	Instructions of the video guide	Execution of each activity (e.g., skipping, jumping)	20s
	Instructions from the video guide	Recovery and explanation of the following activity	10s
		End: reincorporation into the other class activities	30s
CG	This group had their normal school activities in the different subjects without the application of active breaks		

EG, experimental group; CG, control group; min, minutes; s, seconds.

One week before implementing the program, all the teachers were trained for 45 min. A researcher conducted the training sessions in the schools. The training session was designed to instruct teachers on the necessary skills and knowledge to implement ABs. The training session’s content included the importance of adding PA to the classroom routine and instructions for program application. After the training session, digital materials were provided.

### 2.5 Statistical analysis

All statistical analyses were performed using the Jamovi software (53). The data are reported as mean and standard deviation for both EG and CG. Within the PA level factor, differences between the groups’ scores of the EG and CG were verified using an analysis of variance (ANOVA) comparison of the PA level during the entire intervention. For on-task behavior, the statistical differences in percentage before and after ABs were verified using a paired *t*-test. Moreover, for each on-task behavior variable score evaluated after the intervention, the absolute variation (*Δ*) and percentage of variation (Δ%) concerning its pre- and post-intervention values were calculated to produce an acute increase in primary post-intervention values. We considered the results statistically significant only if the *p*-value was less than 0.05. The effect size (ES) was calculated using Cohen’s *d*. Effect sizes of less than 0.4 represented a small difference, whereas effect sizes of 0.41–0.7 and greater than 0.7 represented a moderate or large difference, respectively (54).

## 3 Results

A total of 55 children completed the intervention. No adverse events were reported, and no students were excluded; therefore, data from all the participants were analyzed. There were no significant differences between the groups in terms of age, height, and weight (Table 3).

**Table 3:** Sample characteristics.

Outcome	Groups		*p*-*values*
	EG (*n* = 27)	CG (*n* = 28)	
	Mean ± SD	Mean ± SD	
Age (years)	10.28 ± 0.71	10.23 ± 5.85	0.80
Height (cm)	154 ± 0.07	155 ± 0.06	0.76
Weight (kg)	54.61 ± 11.31	55.57 ± 13.81	0.95

EG, experimental group; CG, control group; cm, centimeters; kg, kilograms.

Table 4 shows the PA data collected during the school day for each student. The EG showed statistical differences in the min of PA level on the five levels: Sedentary Time (ST): The min of the CG on sedentary behavior were significant (EG 229.83 ± 17.17 vs. CG 253.76 ± 12.8 min, *p* = 0.001; ES = −1.58), Light Physical Activity (LPA) (EG 36.65 ± 11.66 vs. CG 32.20 ± 7.77 min, *p* = 0.002; ES = 1.04), Moderate Physical Activity (MPA) (EG8.78 ± 2.98 vs. GC 7.11 ± 1.81 min, *p* = 0.002; ES = 1.05), Vigorous Physical Activity (VPA) (EG14.76 ± 4.83 vs. CG 6.52 ± 3.23 min, *p* = 0.001; ES = 2.64), and Moderate and Vigorous Physical Activity (MPVA) (EG 23.53 ± 7.12 vs. GC 13.71 ± 4.7 min, *p* = 0.001; ES = 2.18). Consequently, the EG showed higher PA levels at different intensities and consequently less sedentary time than the CG. In addition to establishing statistically significant differences in means, it also showed a large effect size, except for the ST category (Table 4).

**Table 4:** Results: physical activity levels.

Outcomes	Groups				*p*-values	ES
	EG (*n* = 27)		CG (*n* = 28)			
	Mean ± SD	IC 95%	Mean ± SD	IC 95%		
ST (min)	229.83 ± 17.17	221.71–237.81	253.76 ± 12.81	247.61–25.91	0.001	−1.58
LPA (min)	36.65 ± 11.66	31.19–42.11	32.20 ± 7.77	22.61–29.91	0.002	1.04
MPA (min)	8.78 ± 2.98	7.38–10.18	7.11 ± 1.81	5.11–7.01	0.002	1.05
VPA (min)	14.76 ± 4.83	12.47–17.04	6.52 ± 3.23	3.82–5.82	0.001	2.64
MVPA (min)	23.53 ± 7.12	20.20–26.86	13.71 ± 4.71	9.11–12.81	0.001	2.18

EG, experimental group; CG, control group; ST, sedentary time; LPA, light physical activity; MPA, moderate physical activity; VPA, vigorous physical activity; MVPA, moderate and vigorous physical activity; min, minutes; ES, effect size.

Regarding on-task classroom behavior outcomes (Table 5), the data showed that both *academic engagement* (67.52 ± 25.61% vs. 82.91 ± 18.81%; *p* = 0.002; ES = 0.1) and *disruptive behavior* (15.81 ± 17.22% vs. 7.5 ± 14.8%; *p* = 0.002; ES = 0.5) were statistically different before and after the ABs in the EG; however, the ES was weak for academic engagement and medium for *disruptive behavior*. In addition, the *Δ* and Δ% change in both variables (*academically engaged*: Δ 0.8, Δ% change +15.4; *disruptive*: Δ 2.1, Δ% change −8.3) clearly show the difference between the pre- and post-ABs. Regarding *respectfulness* (84.21 ± 17.41% vs. 90.41 ± 14%; *p* = 0.21), the ABs did not produce any change. However, the Δ and Δ% change showed modifications (Δ 0.9, Δ% change +6.2).

**Table 5:** Results on-task classroom behavior.

Outcomes	EG (*n* = 27)		Δ % change	Δ values	*p*-values	ES
	Pre-ABs	Post-ABs				
	Mean ± SD	Mean ± SD				
Academically engaged (%)	67.51 ± 25.61	82.91 ± 18.81	+15.4	0.8	0.002	0.1
Respectful (%)	84.21 ± 17.41	90.41 ± 14.01	+6.2	0.9	0.21	0.2
Disruptive (%)	15.81 ± 17.22	7.51 ± 14.81	−8.3	2.1	0.002	0.5

EG, experimental group; ABs, active breaks.

## 4 Discussion

The aim of this study was to determine the acute effect of an AB intervention on PA levels and on-task classroom behavior in schoolchildren. ABs of 4 min 30 s, applied six times during the school day. The results showed a significant acute effect on both study variables.

### 4.1 Acute effect on PA level

The analysis showed a significant increase in PA levels (LP, MPA, VPA, and MVPA) in the EG compared to the CG. Simultaneously, ST decreased, suggesting that ABs had a beneficial effect, as demonstrated in earlier studies (55). This result is especially important since the evidence (56) suggests that the focus and priority should be on identifying strategies to increase PA levels in children and adolescents in schools, as it is one of the primary environments where they spend much of their time. A significant decrease in the total daily PA was observed during the transition from primary to secondary school (57). The age of the participants in this study highlights the need to increase opportunities for adolescents to be physically active, particularly during this transitional period (57, 58). In this line, the present study has shown the acute effects of implementing ABs sessions, reporting an increase in the PA level of the EG. This result agrees with another study conducted on ABs with similar characteristics, lasting between 5 and 10 min, in a population of the same age range (59). Another study (60) shows that the application of ABs and physically active learning has an acute effect on increasing PA levels.

Therefore, the study mentions that these types of resources to incorporate PA in the school context collaborate with the recommendation of practicing 60 min of MVPA in the child population. This corroborates the results of the present study, since one of the findings showed a significant increase in the level of MVPA during the school day. In this sense, a meta-analysis (61) carried out coincides with the results mentioned, indicating that, in light of the results, ABs are a promising alternative to increase the level of PA; however, their effects related to learning should be further investigated. In relation to the above, the evidence is compelling regarding the benefits of implementing ABs as a significant factor in increasing PA levels and the benefits of improving classroom behavior (42) and counteracting health risk factors (62, 63).

### 4.2 Acute effect on on-task classroom behavior

The findings of the present study related to on-task classroom behavior coincide with another study (64), whose results demonstrate that 4-min ABs sessions of FUNterval activities—implemented as high-intensity interval training (52) and similar to the activities used in this study—improve both on-task behavior and PA levels, regardless of the time of day. Performing ABs at any time of the school day is recommended to derive the greatest improvements in on-task behavior across the school day (64), especially for students who are less integrated into school tasks (33). According to this line, one of the first programs related to the implementation of ABs was TAKE10! a program that demonstrated positive acute and chronic effects with 10-min periods of PA in the classroom, promoting greater commitment to schoolwork (65). The evidence has been overwhelming for some years regarding the positive effects of ABs on on-task classroom behavior (66) and their effectiveness in increasing the level of PA (67), which agrees with this study; however, progress is still needed in terms of cognitive benefits (19). Therefore, ABs become a powerful alternative for improving primary school students’ well-being without disrupting their schoolwork (67). However, despite promising results, a systematic review (68, 69) was conducted and analyzed randomized and non-randomized studies and found that the overall available evidence points to a beneficial effect of exercise on attention and on-task behavior in a classroom setting. Nevertheless, methodological differences concerning participants, duration, and type of PA should be considered when comparing the results. Further studies with more comparable methodologies are needed to provide a better understanding of the acute effects of ABs on task behavior. In this sense, this study is one of the first to address the acute effects of ABs on on-task behavior. This line of research needs to be explored further.

## 5 Strengths and limitations

One of the strengths of this study is the use of device-measured PA through accelerometry, which allowed for accurate quantification of acute changes in PA levels with ABs. Another strength is the analysis of task behavior, as it is one of the most important components of learning and one of the topics of greatest interest to teachers. Regarding limitations, the sample size was adequate, though not large, which is common in intervention studies conducted in the educational field. This limitation is further accentuated by the specific measurement characteristics of the on-task classroom behavior outcome, making it even more challenging to recruit a larger number of participants in the study. We acknowledge that a larger sample size could have strengthened the statistical power of our analysis. It is important to mention that it was not possible to blind the data collectors, which could have led to a potential observer bias. Although measures were taken to standardize the observation procedure, such as training the observers, the lack of blinding could have affected the internal validity of the study. Therefore, we recommend that future studies employ a double-blind design to minimize this type of bias and ensure that the results accurately reflect the true effects of ABs.

## 6 Conclusion

ABs address both the time-related barriers teachers encounter when integrating PA into their lessons and reinforce the positive role they play in the learning environment. In summary, the present study demonstrated that ABs of 4 min 30 s are time-efficient, require only basic equipment, can be completed in the classroom, are feasible to implement, and can significantly improve acute PA levels and on-task classroom behavior in schoolchildren. This last finding has enormous practical applications and highlights the importance of integrating PA into learning strategies. It is hoped that these results will help prioritize the inclusion of PA through ABs in school curricula. Implementation should be considered by policymakers and educational authorities.

## Data Availability

The raw data supporting the conclusions of this article will be made available by the authors, without undue reservation.
